# Pediatric low-grade glioma models: advances and ongoing challenges

**DOI:** 10.3389/fonc.2023.1346949

**Published:** 2024-01-22

**Authors:** Griselda Metta Yvone, Joshua J. Breunig

**Affiliations:** ^1^ Board of Governors Regenerative Medicine Institute, Cedars-Sinai Medical Center, Los Angeles, CA, United States; ^2^ Department of Biomedical Sciences, Cedars-Sinai Medical Center, Los Angeles, CA, United States; ^3^ Center for Neural Sciences in Medicine, Cedars-Sinai Medical Center, Los Angeles, CA, United States; ^4^ Samuel Oschin Comprehensive Cancer Institute, Cedars-Sinai Medical Center, Los Angeles, CA, United States; ^5^ Department of Medicine, David Geffen School of Medicine, University of California, Los Angeles, Los Angeles, CA, United States

**Keywords:** pLGG, pediatric low grade glioma, NF1, BRAF, mouse models, preclinical model, *KIAA*-*1549*-fusion, optic glioma

## Abstract

Pediatric low-grade gliomas represent the most common childhood brain tumor class. While often curable, some tumors fail to respond and even successful treatments can have life-long side effects. Many clinical trials are underway for pediatric low-grade gliomas. However, these trials are expensive and challenging to organize due to the heterogeneity of patients and subtypes. Advances in sequencing technologies are helping to mitigate this by revealing the molecular landscapes of mutations in pediatric low-grade glioma. Functionalizing these mutations in the form of preclinical models is the next step in both understanding the disease mechanisms as well as for testing therapeutics. However, such models are often more difficult to generate due to their less proliferative nature, and the heterogeneity of tumor microenvironments, cell(s)-of-origin, and genetic alterations. In this review, we discuss the molecular and genetic alterations and the various preclinical models generated for the different types of pediatric low-grade gliomas. We examined the different preclinical models for pediatric low-grade gliomas, summarizing the scientific advances made to the field and therapeutic implications. We also discuss the advantages and limitations of the various models. This review highlights the importance of preclinical models for pediatric low-grade gliomas while noting the challenges and future directions of these models to improve therapeutic outcomes of pediatric low-grade gliomas.

## 1 Introduction

Pediatric low-grade glioma (pLGG, WHO grade 1 or 2) is the most common brain tumor class in children (1–4). Although in general, prognosis, overall survival, and outcomes following treatment are better for pLGG than adult gliomas, the broad spectrum and heterogeneity of pLGG histopathology make these tumors challenging to treat (1, 3–6). For example, these gliomas may differ in their brain locations, histological spectrum, cell(s) of origin, genetic alterations, and the tumor microenvironment milieu (4). Additionally, since many pLGGs undergo senescence towards juvenile period or adulthood, a lot of pLGG patients end up not getting surgeries, making it harder to obtain tumor samples to be studied. For tumors located in brain areas accessible by surgery such as the cerebellum, total resection is a viable option and may be curative, but for less accessible tumors such as those in hypothalamus, midline areas, and optic pathway, additional treatments such as radiation and cytotoxic chemotherapies may be necessary (1, 7). Even with gross total resection, recurrence may still happen, and additional treatments may lead to allergies and long-term side effects (5, 7, 8). Furthermore, since pLGGs are chronic diseases, these children sustain significant life-long morbidity and general reduction in quality of life. Due to the complexity of the molecular landscape of pLGG, continuous efforts are directed towards characterizing the genomic and epigenomic alterations in these gliomas, and developing effective preclinical models are paramount in making informed decisions on therapeutic options.

## 2 Molecular landscape of pLGG

**Figure 1:**
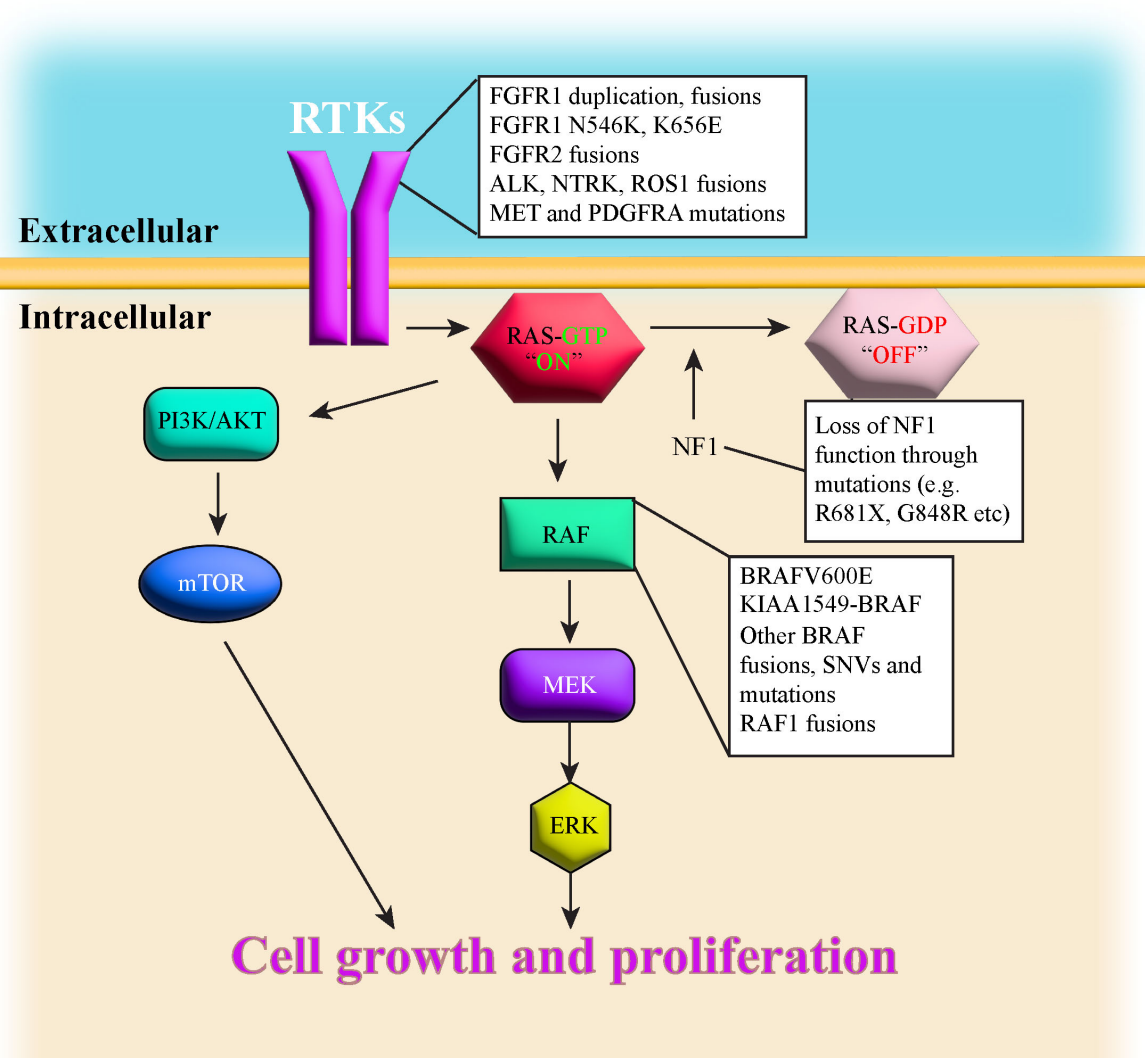
illustrates a simplified schematic of the MAPK signaling pathway. Many components of the MAPK pathway are altered in pLGGs.

Advances in sequencing technologies have aided in classifying the diverse spectrum of pLGG histopathology. The various types of pLGG that are more often encountered are summarized in **Table 1**, including their locations and most common genetic alterations. pLGGs can be glial or mixed glial-neuronal tumors. Pilocytic astrocytomas (PAs) predominate in children younger than 15 years of age and is considered the most common type of pLGG (1, 7). However, it is still very challenging to accurately diagnose and stratify pLGG patients due to the broad spectrum of the histopathology and the complex molecular landscape (1, 4, 9, 14, 18). Moreover, in many cases, there are overlapping morphologies between the different groups of pLGGs, and even the more circumscribed tumors can contain infiltrative areas (4, 9, 14). The majority of pLGGs show convergence in MAPK (Mitogen-activated protein kinase)/ERK (Extracellular signal-regulated kinase) pathway alterations, as illustrated in **Figure 1** and the next section will discuss in more details the genetic alterations found in various pLGGs based on advancement in molecular profiling studies (10, 11, 19).

**Table 1:** pLGGs comprise heterogeneous histopathological and molecular alterations.

Name	Group	WHO grade	Location	Molecular alterations	References
Pilocytic astrocytoma (PA)	Glial tumors/circum-scribed astrocytomas	1	Posterior fossa/cerebellum* but can arise in optic pathway or any part of CNS	KIAA1549-BRAF*, *BRAF* V600E, *NF1*, *FGFR1* hotspot mutations, *NTRK2*, *KRAS*, generally MAPK pathway	Zhang et al., 2013; Garcia et al., 2016; Ryall et al., 2020; Milde et al., 2021; Louis et al., 2021 (1, 4, 9–11)
Pilomyxoid astrocytoma (PMA)	Glial tumors	2	Hypothalamic/chiasmatic region	*KIAA1549-BRAF*, other MAPK pathway alterations	Garcia et al., 2016; Milde et al., 2021 (1, 4)
Pleomorphic xanthoastrocytoma (PXA)	Glial tumors/circumscribed astrocytomas	2 or 3	Typically supratentorial, particularly in temporal lobe	*BRAF* V600E*, *CDKN2A* or *CDKN2B* other MAPK/ERK pathway gene alterations	Zhang et al., 2013; Garcia et al., 2016; Ryall et al., 2020; Milde et al., 2021; Louis et al., 2021 (1, 4, 9–11)
Diffuse astrocytoma (DA)	Diffuse low-grade gliomas	2	Cerebral hemispheres	*BRAF* V600E*, MYB or *MYBL1*, *FGFR1*, *KIAA1549-BRAF*	Zhang et al., 2013; Garcia et al., 2016; Ryall et al., 2020; Louis et al., 2021 (1, 9–11)
Diffuse midline glioma	Glial tumors	2 (may progress to 3 or 4)	Thalamus, pons, spinal cord, midline structures	*H3*-K27M	Garcia et al., 2016 (1)
Subependymal giant cell astrocytoma (SEGA)	Glial tumors/circum-scribed astrocytomas	1	Lateral ventricles	Germline mutations in *TSC1* or *TSC2*	Garcia et al., 2016; Louis et al., 2021 (1, 9)
Low grade oligodendroglioma	Glial tumors	2 or 3	Cerebral hemispheres	FGFR1*; BRAF V600E	Garcia et al., 2016; Ryall et al., 2020 (1, 11)
Dysembryoplastic neuroepithelial tumors (DNET)	Mixed glioneuronal tumors	1	Cerebral hemispheres, typically temporal lobe	*BRAF*V600E, FGFR1*	Garcia et al., 2016; Ryall et al., 2017; Ryall et al., 2020 (1, 11, 12)
Astroblastoma	Glial tumors	Not assigned	Cerebral hemispheres	Unknown	Garcia et al., 2016 (1)
Angiocentric glioma	Mixed glioneuronal tumors	1	Superficial cerebrocortical	*MYB*	Garcia et al., 2016; Jones et al., 2018; Ryall et al., 2020; Louis et al., 2021 (1, 3, 9, 11)
Polymorphous low-grade neuroepithelial tumor of the young (PLNTY)	Mixed glioneuronal tumors	1	Mostly temporal lobe	*BRAF* V600E*; FGFR2*-*CTNNA3* fusion; MAPK/ERK pathway	Bale, 2020; Ryall et al., 2020; Louis et al., 2021 (9, 11, 13)
Anaplastic Pilocytic Astrocytoma (APA)	Glial tumors	1 or 2 (may progress to 3)	Mostly cerebral hemispheres but can arise in any part of CNS	*CDKN2A/B, NF1, ATRX* mutations	Milde et al., 2021 (4)
Extra ventricular neurocytoma (EVN)	Mixed glioneuronal tumors	2	Extraventricu-lar space	FGFR1-TACC1* fusion*, FGFR3-TACC3, FGFR1-EVI5*	Bale, 2020; Louis et al., 2021 (9, 13)
Rosette-forming glioneuronal tumor (RGNT)	Mixed glioneuronal tumors	1	Cerebral hemispheres, midline	FGFR1* hotspot mutations (N546K and K656E), *PIK3CA*, *NF1; KIAA1549-BRAF*	Sievers et al., 2019; Lucas et al., 2020; Bale, 2020; Appay et al., 2022; Louis et al., 2021; Ryall et al., 2020 (9, 13–17)
Ganglioglioma	Mixed glioneuronal tumors	1	Cerebral hemispheres	*BRAF* V600E*; *KIAA1549-BRAF*	Ryall et al., 2020; Louis et al., 2021 (9, 14)
Desmoplastic infantile ganglioglioma and astrocytoma	Mixed glioneuronal tumors	1	Cerebral hemispheres	*BRAF* V600E*; *FGFR1*; *KIAA1549-BRAF*	Garcia et al., 2016; Ryall et al., 2020; Louis et al., 2021 (1, 9, 14)
Papillary glioneuronal tumor	Mixed glioneuronal tumors	1	Cerebral hemispheres	*SLC44A1-PRKCA*	Ryall et al., 2020; Louis et al., 2021 (9, 14)
Chordoid glioma of third ventricle	Mixed glioneuronal tumors	2	Third ventricle	*PRKCA*	Ryall et al., 2020; Louis et al., 2021 (9, 14)
Multinodular and vacuolating neuronal tumor	Mixed glioneuronal tumors	1	Cerebral hemispheres	MAP2K1*; BRAFV600E; *FGFR2* fusions; MAPK pathway	Ryall et al., 2020; Louis et al., 2021 (9, 14)

*indicates the most common location and alteration.

### 2.1 BRAF

B-raf proto-oncogene (*BRAF*) encodes a protein belonging to the RAF family of serine/threonine protein kinases. This protein is part of the MAPK/ERK signaling pathway, which affects cell division, differentiation, transcription, and many other cellular processes (6).

Fusion with *KIAA1549* gene (*KIAA1549-BRAF*) and *BRAF* V600E (Valine to Glutamic Acid) mutation are the most frequent *BRAF* alterations found in pLGGs, with *KIAA1549-BRAF* being almost exclusively a single-event driver (11). Other rarer *BRAF* alterations include fusions with partners other than *KIAA1549* (*FAM131B*, *RNF130*, *CLCN6*, *MKRN1*, *GNA11*, *QKI*, *FZR1*, and *MACF1*) that mainly result in loss of the N-terminal regulatory region of the BRAF protein and retention of the kinase domain (19, 20), insertion at position 600 (V600ins), and single nucleotide variant (SNV) at position 594 (D594N (11). Jones et al. (2013) also found three amino acid insertion (Valine-Leucine-Arginine) that leads to stabilization of a dimeric form of BRAF and increased ERK phosphorylation, equivalent to the *BRAF* V600E mutant (19).

*KIAA1549-BRAF* fusion is the most common alteration found in PA and cerebellar tumors (10, 11, 19). This fusion is characteristic of sporadic juvenile PA (JPA) and does not occur in NF1-PA (PA with mutations in *NF1* gene (21). The fusion occurs due to tandem duplication of *BRAF* gene that results in an in-frame fusion gene incorporating the *BRAF* kinase domain (22). The N-terminal end of the KIAA1549 protein replaces the N-terminal regulatory region of BRAF protein (5’ end of *KIAA1549* gene and 3’ end of *BRAF* gene), leading to constitutive activation of the BRAF kinase domain (20, 22). The most common *KIAA1549-BRAF* fusion involved exons 1-16 of *KIAA1549* gene and exons 9-18 of *BRAF* gene (16:9), although fusion between *KIAA1549* exon 15 and *BRAF* exon 9 (15:9) was observed in hemispheric tumors and was associated with a worse progression-free survival compared with other fusions (11, 20, 22). A few infants with 15:11 fusion rapidly progressed and died (11).

*BRAF* V600E, another common mutation found in PAs, is often associated with additional alterations such as deletion in the tumor suppressor gene *CDKN2A* (Cyclin dependent kinase inhibitor 2A), SNVs in *NF1*, *FGFR1* (Fibroblast growth factor receptor 1), *KRAS* (Kirsten rat sarcoma viral oncogene homolog), and *H3F3A* (H3 histone family member 3A), but never with fusion events. Interestingly, the combination of *BRAF* V600E and *CDKN2A* loss likely leads to tumor transformation and higher tumor grade (12). *BRAF* V600E mutation is found in multiple types of pLGGs such as ganglioglioma, diffuse astrocytoma (DA) and Pleomorphic Xanthoastrocytoma (PXA). *BRAF* V600E-driven pLGG most frequently occurs in the cerebral hemispheres and diencephalon (11).

Ryall et al. (2020) found that patients with *KIAA1549-BRAF* fusion have better outcome compared to those with *BRAF* V600E mutation in terms of progression-free survival. However, patients with *BRAF* V600E coupled with *CDKN2A* deletion tend to progress and succumb to their disease (11).

### 2.2 NF1

Patients with Neurofibromatosis type 1(NF1) suffer from a dominantly inherited genetic disease. These patients develop benign tumors, termed neurofibromas, along the nerves of body (23). Neurofibromatosis type 1 is considered as one of the most common genetic disorders in humans (7). Neurofibromin 1, the protein product of the *NF1* gene, is a negative regulator of RAS (Rat sarcoma virus) in the MAPK/ERK pathway as it mediates the conversion of activated Ras-GTP to inactive Ras-GDP [ (24–27), **Figure 1**]. Loss of *NF1* activity leads to hyperactivation of downstream RAS effectors (28), thus *NF1* is considered a tumor suppressor gene. NF1-associated astrocytomas usually sustain germline mutation of the *NF1* gene located on chromosome 17q and somatic loss of the remaining *NF1* allele, resulting in bi-allelic inactivation and loss-of-function to the *NF1* gene (7, 8, 29, 30). Children with NF1 are predisposed and may develop PAand optic pathway glioma (OPG (7, 10, 11, 23, 31), although they can also develop DA (8). NF1-associated PAs are most often found in the optic pathway or hypothalamus, whereas non NF1-PA are usually located in cerebellum (7). Sporadic OPGs tend to progress, are more aggressive and usually require treatment, but most NF1-associated OPGs grow slowly, are more indolent and thus don’t require treatment (8). NF1-associated OPGs are usually presented in early childhood and rarely continue to grow or cause symptoms after age 10 (32, 33). In general, the prognosis for children with NF1-associated LGGs is good, as most of these tumors are asymptomatic and require no therapeutic intervention. Additionally, children with NF1-LGGs who receive therapy tend to have better progression-free survival than their sporadic counterparts (8).

### 2.3 FGFR

The Fibroblast Growth Factor Receptor (FGFR) family consists of four transmembrane tyrosine kinase receptors (FGFR1-4) that dimerize in response to ligands, triggering downstream pathways including MAPK and phosphatidylinositol-3-kinase (PI3K)/AKT (Protein Kinase B) pathways implicated in tumorigenesis (13). FGFR signaling plays a role in angiogenesis, tumor cell migration, differentiation, proliferation and survival (13).

There are multiple types of *FGFR1* alterations described in pLGGs including tyrosine kinase domain duplication (*FGFR1* TKDD), fusions (*FGFR1-TACC1*), and *FGFR1* hotspot mutations (N546K and K656E). *FGFR1* TKDD appears to be largely absent in high-grade gliomas (HGG (10), but it’s been reported in rosette-forming glioneuronal tumor (RGNT), anaplastic PA, glioneuronal tumor with PA and PXA features (34, 35). *FGFR1* TKDD includes exons 10-18, producing an in-frame fusion and duplicates the entire *FGFR1* region encoding tyrosine kinase domain (10, 13, 36).

Meanwhile, *FGFR1-TACC1* fusions have been reported in extraventricular neurocytoma (EVN) and in cerebral hemispheric PA (10, 13). Other fusion events involving *FGFR1* (37) and *FGFR2* (e.g. *FGFR2-CTNNA3*) have also been found in polymorphous low grade neuroepithelial tumor of the young (PLNTY (13). Other *FGFR2* fusions reported include *FGFR2-INA* and *FGFR2-ERC1* (11).

*FGFR* fusions or duplications usually lead to constitutive FGFR activity and activation of downstream pathways such as MAPK/PI3K/mechanistic target of rapamycin (mTOR (10, 12, 13, 19). *FGFR1* TKD and *FGFR2* fusions predominate in glioneuronal or oligodendroglial tumors and mostly occur in the cerebral hemispheres (11, 13).

Patients with *FGFR1-TACC1* fusion or TKD duplications tend to have better progression-free survival compared to patients with *FGFR1* SNVs (11).

*FGFR1* hotspot mutations mainly consist of N546K (Asparagine to Lysine) and K656E (Lysine to Glutamic acid). These mutations are most commonly found in midline tumors such as dysembryoplastic neuroepithelial tumors (DNETs (10, 19, 38–40), but they have been reported in posterior fossa PA with widespread oligodendroglial features (15), extracerebellar PA (19), RGNTs (16, 17) and diffuse midline gliomas along with H3K27M mutations (41). These *FGFR1* hotspot mutations can also co-occur with other genetic alterations including *NF1*, other *FGFR1* point mutations or other RAS/MAPK pathway alterations (10, 13).

It appears that while pLGG patients with *FGFR* gene family alterations may progress and have worse outcome, the tumors rarely result in deaths (11, 42).

### 2.4 MYB

The *MYB* (Myeloblastosis) gene family consists of *MYB*, *MYBL1* (*MYB* proto-oncogene like 1), and *MYBL2*, encoding the transcription factors MYB (c-MYB), MYBL1 (A-MYB), and MYBL2 (B-MYB (43). Since MYB proteins are essential for cellular growth, differentiation, and survival, they have been found to be aberrantly expressed in human cancers (43).

Tatevossian et al. (2010) reported alterations in *MYB/MYBL1* in pediatric diffuse glioma (44). Rearrangements and copy number abnormality in *MYB* or *MYBL1* resulting in upregulated MYB or MYBL1 are found in more infiltrative pLGGs including grade 2 DAs, angiocentric gliomas and oligodendroglioma (10, 44, 45). *MYB* alterations are associated with a deletion of a 3’ portion of *MYB* gene, involving either the negative regulatory region or 3’ UTR microRNA binding sites (10). Bandopadhayay et al. (2016) found a novel *MYB-QKI* (K-homology domain containing RNA binding) fusion event in angiocentric glioma (46). Interestingly, no *MYB* alterations are identified in pediatric HGGs or ependymomas (10). Although progressions are rare in *MYB*-altered tumors, they were more frequent in *MYBL1*-altered tumors (11).

### 2.5 Other mutations: NTRK2, TSC, H3K27M, KRAS

Fusions in other Receptor Tyrosine Kinases (RTKs) are rare in pLGGs and may include *ALK* (Anaplastic lymphoma kinase) gene fusions (*CCDC88A-ALK*, *PPP1CB-ALK*), *ROS1* (ROS proto-oncogene) fusions (*GOPC-ROS1*), *NTRK2* (Neurotrophic tyrosine receptor kinase)*/TRKB* (Tropomycin receptor kinase B) fusions (*NTRK2-MID1*, *NTRK2-SF3B1*) and *PDGFB* fusions (*PDGFB-LRP1*). These fusions mainly occur in cerebral hemispheric tumors, although *ROS1* fusions are also seen in intraventricular space. Patients with these alterations rarely progress and/or succumb to their diseases (11).

Alterations in other members of the RAS/MAPK pathway have been documented in pLGG cases including *RAF1* fusions, *KRAS* mutations, and *MAP2K1* deletions (11).

Some pLGG patients also exhibit alterations in other RTKs including mutations in MET or *PDGFRA*, *ALK*, *NTRK2* (*TRKB*). Jones et al. (2013) identified *QKI-NTRK2* and *NACC2-NTRK2* fusions in pLGGs, resulting in ligand-independent dimerization and indirectly activation of MAPK pathway (19).

Another small percentage of patients have *H3F3A* mutations (11), and it is most often K27M. The tumors are restricted to the midline and brainstem and tend to be DAs. H3K27M also often co-occurs with other alterations, most often with *BRAF* V600E. These patients tend to progress early (11).

*IDH1* R132H mutations, while very common in adult lower-grade gliomas, are extremely rare in pLGGs. These tumors tend to occur in the cerebral hemispheres and are either oligodendroglioma or diffuse astrocytoma, and the patients may progress (11).

## 3 *In vitro* models of pLGG: the path to establishing patient-derived pLGG cell lines for preclinical testing

**Figure 2:**
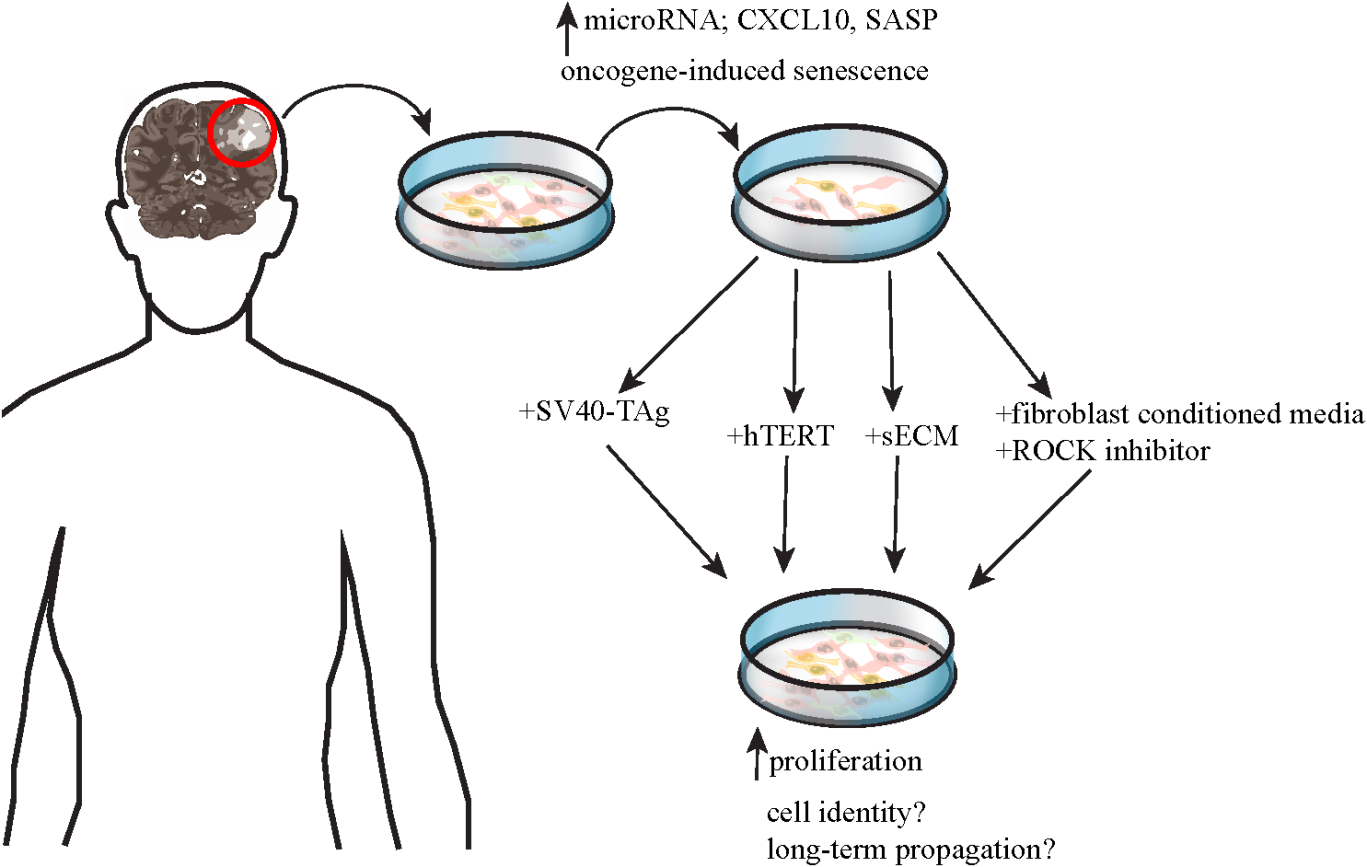
shows a schematic of the efforts made to establish patient-derived pLGG cell lines for preclinical testing, including OIS phenomenon which represents a major challenge to these endeavors. Factors like microRNAs, CXCL10, and SASP have been implicated in OIS. Several methods have been developed to extend lifespan of the pLGG cells in culture to varying degree of success and with remaining concerns on whether the cultured cells represent the original patient tumors and whether the cells can be stably propagated long-term for use in preclinical testing.

As sequencing technologies advance, more mutations are continuously discovered in various types of pLGGs, complicating their molecular landscape. Moreover, different mutations may cause the different tumors to respond variably to targeted therapeutic agents, making risk stratification and development of therapies for pLGG difficult. Efforts to develop preclinical models of pLGGs are mainly focused on the more common molecular alterations including but not limited to *KIAA1549-BRAF*, *BRAF* V600E, and *NF1* mutations, but many have not been successful. **Table 2** lists the *in vitro* models that have been developed for preclinical testing and their varying degrees of success. One obstacle in generating viable pLGG patient-derived cell lines is the lack of availability of patient tissues. Tumors located in areas difficult to surgically excise are often not biopsied, making it difficult to obtain sufficient samples from these patients to generate cell lines (4). Another major obstacle in generating viable cell lines for non-NF1-pLGG (e.g. PA) is the oncogene-induced-senescence (OIS) phenomenon [ (48, 49), **Figure 2**]. Expression of *BRAF* V600E and *KIAA1549-BRAF* fusion led to MAPK pathway activation and subsequent OIS in human cortical neurospheres, human immortalized astrocytes, fetal astrocytes, and low-passage primary PA cultures [ (48, 49), **Table 2**]. The OIS phenomenon, however, may be the rationale for the lack of progression of PA to higher-grade tumors, in the absence of additional cooperating mutations (48, 49). Factors like senescence-associated secretory phenotype (SASP) and microRNAs have been implicated in senescence of pLGG cell lines (58, 59). On the other hand, inactivation of *CDKN2A* gene encoding the tumor suppressor p16INK4a might facilitate escape from senescence in these cells, explaining the association between p16INK4a loss and worse outcomes in PA patients (48, 49).

**Table 2:** List of *in vitro* models developed for preclinical testing.

Name/Type of model	Source/Mutations	Caveats/Notes	Reference
Patient-derived cell lines (Res259 and Res186)	Grade 2 DA and Grade 1 PA	Might harbor additional genetic alterations	Bax et al., 2009 (47)
Human immortalized and fetal astrocytic cell lines	Overexpression of WT and *BRAF* V600E	Low passage due to oncogene-induced senescence	Jacob et al., 2011 (48)
Human neural stem and progenitor cells and neurospheres	Overexpression of *BRAF* V600E	Low passage due to oncogene-induced senescence	Raabe et al., 2011 (49)
Patient-derived primary cells	*KIAA1549-BRAF*-expressing PA	Low passage due to oncogene-induced senescence	Raabe et al., 2011 (49)
Patient-derived cell lines (BT-35 and BT-40)	Juvenile PA with 2-5 copies of WT *BRAF* and 5 copies of *BRAF* V600E	Might harbor additional genetic alterations resembling higher-grade tumors	Kolb et al., 2010; Bid et al., 2013 (50, 51)
Patient-derived cells	PA	Could not form 3D spheroids or be cultured for more than 5 passages as monolayer cells	Sanden et al., 2015 (52)
Patient-derived cell line (DKFZ-BT66)	*KIAA1549-BRAF*-expressing PA	Expressing SV40 large T antigen to propagate better in culture	Selt et al., 2017 (53)
Patient-derived primary cells	Grade 1 pLGG tumors: Infratentorial PA, infratentorial ganglioglioma, supratentorial DNET, supratentorial angiocentric glioma, supratentorial ganglioglioma	Underwent senescence after 30 days of culture, but were not genetically modified	Chiacchiarini et al., 2021 (54)
Patient-derived cells (hTERT-LGG2)	PA with WT *BRAF*	Overexpressed hTERT to extend lifespan of monolayer culture, insufficient to induce immortalization	Franzese et al., 2021 (55)
Patient-derived cells with fibroblast conditioned media and ROCK inhibitor	NF1-associated PAs and pLGGs expressing *BRAF* V600E	Cultured up to 27 passages, some might have acquired additional mutations in culture	Yuan et al., 2021 (56)
Patient-derived cells with synthetic ECM co-culture	Various pLGGs with mutations in *NF1*, *KIAA1549-BRAF*, *BRAF* V600E	Cultured for up to 1 month ex-vivo but could not propagate stable cell lines from these cultures	Rota et al., 2022 (57)

Attempts to generate pLGG patient-derived cell lines yielded varying success rates. Most of them could only be maintained as short-term cultures [ (52, 54, 60), **Table 2**] and/or they acquired additional alterations in culture that made them unrepresentative of the original tumors (47, 50). Bax et al. (2009) conducted molecular and phenotypic characterization of Res259 and Res186 cell lines derived from human pediatric astrocytomas to incorporate them in preclinical testing. Res259 and Res186 were derived from DA and PA patients, respectively. Although their immunophenotypes closely resembled low-grade lesions, both lines harbored genetic alterations reminiscent of higher-grade gliomas [ (47), **Table 2**].

As part of the Pediatric Preclinical Testing Program (PPTP), 2 astrocytoma cell lines were generated, BT-35 cells which had 2 to 5 copies of wild-type (WT) *BRAF* and BT-40 cells which had 5 copies of activated *BRAF* V600E [ (50, 51), **Table 2**)]. BT-40 was further characterized as grade 2-3 astrocytoma as these cells lost *CDKN2A* and wild-type *P53* (50, 54, 61).

An important milestone in the development of patient-derived PA cell lines was when Selt et al. (2017) transduced cells derived from a 2-year-old PA patient with Dox inducible system coding for simian vacuolating virus 40 large T antigen (SV40-TAg, **Figure 2**). Expression of SV40-TAg inhibited 2 pathways critical for OIS induction and maintenance and enabled the generation of long-term PA cell line which could be especially useful for preclinical drug testing. This *KIAA1549-BRAF*-expressing cell line was named DKFZ-BT66 [ (53), **Table 2**)].

Chiacchiarini et al. (2021) reported additional pLGG cellular models which included primary cells derived from Grade I pLGG tumors. 3 of 9 tumors were infratentorial PAs, 1 of 9 tumors was infratentorial ganglioglioma, 3 of 9 tumors were supratentorial DNETs, and 1 of 9 was supratentorial angiocentric gliomas, and 1 of 9 was supratentorial ganglioglioma (54). Although these patient-derived cells also underwent senescence after about 30 days of culture, they were not genetically modified and thus were more representative of the tumors they were derived from [ (54), **Table 2**)]. Hence, these cells could be useful for short-term *in vitro* experiments.

The overexpression of hTERT (human telomerase reverse transcriptase, the catalytic subunit of telomerase) was proposed to counteract OIS in pLGG cell lines so they regain their proliferative potential for long-term cultures [ (55), **Figure 2**)]. Another method to extend lifespan of pLGG cell lines was proposed by Yuan et al. (2021). Instead of using feeder layer of 3T3 fibroblasts as first described (62), Yuan et al. (2021) co-cultured the pLGG cell lines in conditioned media from irradiated 3T3 cells and Rho kinase (ROCK) inhibitor Y-27632 [ (56), **Figure 2**)]. They found that these culture conditions led to reversible blockage of senescence, increased proliferation, and allowed these cell lines to propagate longer in culture while still maintaining signature genetic changes of the original tumors (56). This method was tried on multiple pLGG cell lines including 4 PAs, 2 gangliogliomas, 2 anaplastic gliomas, 1 anaplastic PXA and others. One cell line derived from NF1-PA (JHH-NF1-PA1) and another from *BRAF* V600E anaplastic PXA (JHH-PXA1) patients exhibited growth sufficient for preclinical testing *in vitro*. More recently, Rota et al. (2022) developed an ex vivo culture system that utilized synthetic extracellular matrices (sECMs) to partially mimic properties of extracellular matrix *in vivo*. This culture method promoted proliferation and enabled propagation of several patient-derived pLGG cells for up to 1 month ex vivo (57). Continuous efforts are made to generate more pLGG patient-derived cell lines that can be cultured long enough for preclinical testing and still resemble the original tumors, but these studies have made major advancements in the field.

## 4 *In vivo* models of pLGG

### 4.1 Generating non-NF1 pLGG xenograft models for preclinical drug testing

One of the ultimate goals for developing pLGG mouse models is for preclinical drug testing. The two patient/mouse heterografts of JPA generated by PPTP, BT-35 and BT-40, had WT *BRAF* and *BRAF* V600E mutation, respectively (50, 51). AZD6244 (Selumetinib), a MEK1/2 inhibitor showed efficacy in BT-40 xenografts, highlighting the MEK signaling pathway as a potential therapeutic target (51). However, although BT-40 xenografts were highly sensitive to Selumetinib and regressed completely during 6 weeks of treatment, the tumors regrew after treatment was stopped (50). Bid et al. (2013) selected and transplanted the resistant tumor clones into Severe Combined Immunodeficient (SCID) mice and resumed the Selumetinib treatment and showed that the resistance of BT-40 xenografts to Selumetinib was mediated by activation of STAT3 signaling, so STAT3 activation could be compensating for MEK inhibition to maintain proliferation and survival (50). Additionally, several studies have documented paradoxical activation of MAPK pathway during preclinical testing or clinical trials of BRAF inhibitors in pLGGs (63, 64). The studies by Kolb et al. (2010) and Bid et al. (2013) generated some of the few xenografts of pediatric low-grade astrocytomas available at that time. However, many of these models do not completely recapitulate patient tumors as they acquired additional genetic alterations, thus emphasizing the need to develop more pLGG *in vivo* models for more relevant preclinical testing. **Table 3** lists the *in vivo* models that have been generated for non-NF1 pLGG and their contributions to elucidating the complexity of non-NF1 pLGG pathogenesis.

**Table 3:** List of *in vivo* models developed for non-NF1 pLGG.

Organism	Type of model	Genotype/Mutation	Cell type specificity	Timing specificity	Cooperating pathways	Region specificity	References
Mouse	Juvenile PA xenograft	WT *BRAF* (BT-35)			✓		Kolb et al., 2010; Bid et al., 2013 (50, 51)
Mouse	Juvenile PA xenograft	*BRAF* V600E (BT-40)			✓		Kolb et al., 2010; Bid et al., 2013 (50, 51)
Mouse	PA xenograft	*BRAF* V600E			✓		Gronych et al., 2011 (65)
Mouse	PA orthotopic xenograft	*KIAA1549-BRAF*	✓		✓	✓	Kaul et al., 2012 (66)
Mouse	Transgenic	*KIAA1549-BRAF*;*BLBPCre*	✓	✓		✓	Kaul et al., 2013 (67)
Mouse	Transgenic	*KIAA1549-BRAF*;*GFAPCre*	✓	✓		✓	Kaul et al., 2013 (67)
Mouse	Transgenic	*KIAA1549-BRAF;NG2 Cre*	✓	✓		✓	Kaul et al., 2013 (67)
Mouse	PXA Orthotopic xenograft	*BRAF* V600E			✓		Kogiso et al., 2017 (60)
Mouse	pLGG Xenograft	Cerebella injection of *KIAA1549-BRAF*-expressing iNPCs, iGRPs, iOPCs into *Rag1^-/-^* mice	✓		✓		Anastasaki et al., 2022 (68)
Mouse	pLGG Xenograft	Cerebella injection of *KIAA1549-BRAF-*expressing astrocytes differentiated from iNPCs	✓				Anastasaki et al., 2022 (68)

Interestingly, intracranial overexpression of full-length *BRAF* V600E could not induce glioma formation alone, but a higher-grade glioma was formed with the additional loss of *Ink4a/Arf* or AKT activation (69). Consequently, Gronych et al. (2011) used the replication-competent avian leukosis virus with splice acceptor/Tv-a (RCAS/Tv-a) system to introduce different *BRAF* constructs into Nestin-expressing neural progenitor cells and found that while the full-length *BRAF* V600E did not induce glioma *in vivo*, the transgenic expression of only the *BRAF* V600E kinase domain in the cerebral hemispheres was sufficient to induce PA formation (65). Additionally, these tumors had MAPK activation and histopathological features reminiscent of those in PA patients (65).

A mouse model expressing *KIAA1549-BRAF* fusion was developed by Kaul et al. (2012) to investigate the role of fusion BRAF protein in sporadic PA (66). They first transduced *KIAA1549-BRAF* into cerebellar neural stem cells (NSCs) and injected these NSCs into the cerebella of 3-wk-old wild-type mice. They observed glioma-like lesions at 6 months post injection (66). Remarkably, *KIAA1549-BRAF* expression increased proliferation of third ventricle and brainstem NSCs, while not really affecting NSCs in the lateral ventricle and neocortex (66). They also found that the regulation of NSC proliferation by *KIAA1549-BRAF* was mediated by mTOR signaling (66).

Subsequently, Kaul et al. (2013) generated a conditional and regulatable *KIAA1549-BRAF* transgenic mouse strain (67). Mice expressing *Lox-STOP-Lox-KIAA1549-BRAF* with tetracycline-responsive (Tet-off) element were generated and intercrossed with Cre-transgenic mice in which Cre was expressed in Brain lipid binding protein (BLBP+ (70), Neuron glia-antigen 2 (NG2+ (71) and Glial fibrillary acidic protein (GFAP+ (72); cells. These led to the expression of *KIAA1549-BRAF* in NSCs starting at E9.5 (*f-BRAF^BLBP^*), neuroglial progenitor cells starting at E14.5 (*f-BRAF^NG2^*), and astroglial progenitor cells starting at E14.5 (*f-BRAF^GFAP^).* Corroborating their previous finding that ectopic *KIAA1549-BRAF* expression specifically increased NSC proliferation (66), *KIAA1549-BRAF* expression was highest in BLBP+ cells, and transgene expression was highest in the cerebellum of *f-BRAF^BLBP^* mice (67). Additionally, there was more *KIAA1549-BRAF* mRNA expression in astrocytes in the cerebellum compared to those in forebrain or brainstem (67). These conditional *KIAA1549-BRAF* mouse models will be useful tools in further interrogation of the spatial, temporal, and cell-type specificity of *KIAA1549-BRAF* expression. Interestingly, Chen et al. (2019) demonstrated that microglia recruitment was required for glioma-like lesion formation *in vivo* following injection of *KIAA1549-BRAF*-expressing cerebellar NSCs (73).

Although attempts to establish neurospheres and monolayer cultures from patient tumors failed due to low yields of tumor cells from patient samples, Kogiso et al. (2017) generated 1 pLGG patient-derived orthotopic xenograft (PDOX) out of 25 mice intracranially implanted with different cerebellar and cerebral pLGGs (60). This PDOX model, designated as IC-3635PXA, was confirmed to be a PXA as it still had low proliferation index without necrosis or aplasia, but it was moderately cellular and infiltrative (60). IC-3635PXA was then serially sub-transplanted in mouse brains four times, but by the third passage, the tumor began developing higher grade features, and by the fourth passage, it transformed into a high-grade tumor (60). Interestingly, *BRAF* V600E mutant allele frequency increased as IC-3635PXA was serially passaged, along with increasing trisomy 9, *CDKN2A* deletion, loss of GFAP and gain of Vimentin expression (60). Therefore, the PDOX model developed by Kogiso et al. (2017) had tumor progression resembling that of the original patient tumor and gave insights on the cellular drivers of tumor progression and molecular changes that occurred as a grade II PXA transformed into a higher-grade tumor.

### 4.2 NF1-LGG genetically engineered mouse models

Since NF1-associated LGG is also a very common group of pLGG, a lot of efforts were made to generate mouse models that recapitulate tumors seen in NF1 patients histopathologically and molecularly. **Table 4** lists the various *in vivo* NF1 pLGG mouse models. Germline *NF1* knockout mice (*NF1* ^-/-^) were embryonic lethal, while *NF1* heterozygous (*NF1^+/-^*) mice did not develop astrocytomas despite having increased astrocyte proliferation *in vivo* (74, 75). Conditional inactivation of *NF1* in neurons (*NF1^SynI^KO*) led to astrogliosis, but the mice did not develop astrocytomas (76). Bajenaru et al. (2002) first developed astrocyte-specific *NF1* conditional knockout mice using Cre/loxP technology in which Cre was expressed in astrocytes by E14.5, and the astrocytes in these mice were *NF1* null (*GFAPCre*;*NF1^flox/flox^* or *NF1^GFAP^CKO*), but these mice also didn’t develop astrocytomas (72). Bajenaru et al. (2003) then generated another type of astrocyte-specific *NF1* conditional knockout mice by first breeding *NF1^+/-^* mice with *NF1^flox/flox^* mice, and then breeding the progenies with *GFAPCre* mice to generate *GFAPCre*; *NF1 ^flox/mut^* (*NF1+/-^GFAP^CKO*) mice. The development of optic nerve gliomas in these mice showed that astrocytoma formation requires *NF1* heterozygosity and loss of *NF1* in astrocytes and highlights this mouse model as one of the few preclinical models available for NF1-associated optic glioma (77). Zhu et al. (2005) described another mouse model for NF1-associated optic pathway glioma by crossing human *hGFAPCre* mice to *NF1^flox/-^* and *NF1^flox/flox^* mice to generate *NF1^hGFAP^CKO* mice. These mice exhibited hyperproliferation of glial progenitor cells, resulting in increased GFAP-expressing astrocytes in developing and adult brains (78). The optic nerve lesions in both mouse models lacked some common features of PA but displayed some morphological and pathological features reminiscent of the NF1-associated human tumors (78). The *NF1^hGFAP^CKO* mice exhibited fully penetrant glial cell hyperplasia and had more severe symptoms of optic pathway gliomas compared to *NF1+/-^GFAP^CKO* mice, possibly due to the timing of Cre activation, and hence loss of *NF1* (E10.5 for *NF1^hGFAP^CKO* vs. E14.5 for *NF1+/-^GFAP^CKO* mice (77, 78). Dasgupta et al. (2005) also showed that activating *KRAS* specifically in astrocytes in *NF1^+/-^* mice similarly led to optic pathway glioma formation (79). Another NF1 mouse model was generated by inactivating *NF1* in neuroglial progenitors starting at E9.5 (*BLBPCre*; *NF1 ^flox/flox^* (70);. These mice exhibited increased neural stem cell proliferation and glial lineage differentiation and eventually developed optic glioma by 3 months (70, 82). Furthermore, Solga et al. (2014) generated genetically engineered mice with *NF1 *loss in NG2+ progenitor cells, which gave rise to oligodendrocytes and astrocytes *in vivo*, but these mice did not develop optic glioma (81). These mouse models emphasize the dependency of NF1-associated optic pathway glioma on timing of *NF1 *inactivation, cell-of-origin, and on the tumor microenvironment as *NF1 *heterozygosity was required for optic glioma formation.

**Table 4:** List of *in vivo* NF1 LGG mouse models.

Organism	Type of model	Genotype/mutation	Optic glioma formation?	Cell type specificity	Timing specificity	Cooperating pathways/microenvironment	Region specificity	References
Mouse	Transgenic	*NF1^+/-^*	No					Brannan et al., 1994; Jacks et al., 1994 (74, 75)
Mouse	Transgenic	*NF1^SynI^KO (SynICre; NF1^flox/flox^ & SynICre; NF1^flox/KO^)*	No	✓				Zhu et al., 2001 (76)
Mouse	Transgenic	*NF1^GFAP^CKO* (*GFAPCre; NF1 ^flox/flox^*)	No	✓				Bajenaru et al., 2002 (72)
Mouse	Transgenic	*NF1+/-^GFAP^CKO* (*GFAPCre*; *NF1^flox/mut^*)	Yes	✓		✓		Bajenaru et al., 2003 (77)
Mouse	Transgenic	*NF1^hGFAP^CKO* (*hGFAPCre; NF1^flox/flox^ & hGFAPCre; NF1^flox/mut^)*	Yes	✓	✓	✓		Zhu et al., 2005 (78)
Mouse	Transgenic	*NF1^+/-^; KRAS^GFAP^*	Yes	✓		✓		Dasgupta et al., 2005 (79)
Mouse	Transgenic	*NF1^BLBP^* CKO *(BLBPCre; NF1^flox/flox^*)	Yes	✓	✓	✓	✓	Hegedus et al., 2007; Lee et al., 2010 (70, 80)
Mouse	Transgenic	*NG2Cre; NF1 ^flox/mut^*	No	✓	✓			Solga et al., 2014 (81)
Mouse	Transgenic	*Olig2Cre; NF1^flox/mut^*	Yes	✓				Solga et al., 2017 (82)
Mouse	Transgenic	*Prom1Cre^ER^; NF1^flox/mut^*	Yes	✓	✓			Solga et al., 2017 (82)
Mouse	NF1 OPG Xenograft	Brainstem injection of o-GSCs into *NF1^+/-^* mice	Yes			✓		Chen et al., 2015 (83)
Mouse	NF1 OPG Xenograft	Brainstem injection of *NF1*-null iNPCs, iGRPs, iOPCs into *Rag1^-/-^* mice	Yes	✓		✓		Anastasaki et al., 2022 (68)
Mouse	NF1 OPG Xenograft	Brainstem injection of *NF1*-null astrocytes differentiated from iNPCs	No	✓				Anastasaki et al., 2022 (68)

The generation of these NF1-pLGG mouse models paved the way for numerous investigations into multiple aspects of NF1-associated OPG including the pathways affected by loss of *NF1* (79, 84, 85), the role of microenvironment in regulating these tumors (83, 86–90), the remarkable specificity of timing, region, and cell-of-origin of NF1-OPG (70, 80–82, 91–93). Importantly, the mouse models enable the preclinical testing of potential therapeutic compounds for this disease (94).

#### 4.2.1 NF1-associated OPG displays temporal and spatial specificity and is highly dependent on cell-of-origin and MAPK pathway activation

The mTOR pathway had been well-implicated in NF1-associated disorders (85, 95, 96), and it was shown that MAPK pathway activation in *NF1*-deficient astrocytes resulted from RAS hyperactivation (78, 85). Dasgupta et al. (2005) showed that activating *KRAS* in astrocytes of *NF1^+/-^* mice was sufficient for the formation of NF1-OPG (79). They further demonstrated that *NF1*-deficient astrocytes exhibited high levels of mTOR pathway activation, and this was inhibited by blocking KRAS or PI3K.

Additionally, optic glioma formation was dependent on the type of germline *NF1* mutation sustained as the nonsense mutation R681X resulted in greater reduction of Neurofibromin level and more proliferative optic glioma compared to the missense mutation G848R (97). Subsequently, the loss of Neurofibromin led to increased ERK, AKT, and in turn, mTOR activation, to drive *NF1*-deficient astrocyte proliferation *in vitro* and NF1 optic glioma growth *in vivo* (97). Furthermore, it became apparent that Neurofibromin regulation of mouse astrocyte and optic glioma growth was mediated by MEK and AKT signaling that all converged on the mTOR complex (96). These studies elucidated the role of MAPK and mTOR signaling in NF1-OPG.

Mouse OPG tumors were found to contain some neoplastic astrocytes that retained markers of astroglial progenitors such as nestin, BLBP, and contained GFAP- and Olig2-immunoreactive cells (86, 98). To investigate the possibility that these neural stem/progenitor cells gave rise to NF1-OPG, Dasgupta and Gutmann (2005) examined the relationship between Neurofibromin and NSCs. Inactivation or loss of *NF1 *led to hyperactivation of RAS, MAPK and AKT, and increased proliferation and survival of NSCs and facilitated engraftment and survival of NSCs *in vivo* (99). Remarkably, NSCs responded differently to *NF1* inactivation depending on the brain regions they belonged to, such that *NF1* loss led to increased NSC proliferation and gliogenesis in the brainstem but not in the cortex (80). This regional specificity in the response of NSC to *NF1 *loss was mediated by AKT and mTOR, as the expression of Rictor, an mTOR complex protein, was higher in brainstem compared to cortex (80). This differential Rictor expression in turn led to region-specific mTOR/Rictor-mediated AKT phosphorylation (80). Interestingly, in astrocytes, mTOR regulated cell growth by activating Rac1 instead of AKT (80, 84). Importantly, Lee et al. (2012) subsequently demonstrated that pediatric optic glioma in *NF1+/-^GFAP^CKO* mice (77) arose from third ventricle as NSCs from this region were the cells that hyperproliferated in response to mutations characteristic of pediatric glioma, and not NSCs from the lateral ventricle subventricular zone (92).

However, third ventricle NSCs were not the only cell population that can serve as initiating population of NF1-OPG. Subsequent research showed that *NF1 *loss in Olig2+ cells (*Olig2Cre*; *NF1^flox/mut^*), which also gave rise to astrocytes in murine optic nerve, also formed optic gliomas, albeit at 6 months (82) instead of 3 months as in *NF1+/-^GFAP^CKO* mice (77). Since in *Olig2Cre* mice, Cre recombinase was expressed by E12, this delay in optic glioma formation was likely due to cell-of-origin and not due to timing of *NF1* loss. Since BLBP+ and GFAP+ neuroglial progenitor cells co-expressed CD133, a neural progenitor/stem cell marker, Chen et al. (2015) investigated whether CD133+ cells could serve as initiating cells for NF1-OPG. They first isolated CD133+ cells, which were characterized to be multipotent low grade optic glioma stem cells (o-GSCs) from tumor-bearing *NF1+/-^GFAP^CKO* mice (83). The transplantation of these o-GSCs into the brainstems of 3-week-old *NF1^+/-^* mice yielded optic gliomas within 6 months, but not transplantation of o-GSCs into brainstems of immunocompromised athymic mice, emphasizing the need for *NF1^+/-^* local microenvironment in glioma formation (83). Solga et al. (2017) generated inducible *NF1* conditional knockout mice in which somatic *NF1 *was eliminated in CD133+ neural progenitor/stem cells at E15 (*Prom1CreER*; *NF1^flox/mut^*). The injection of tamoxifen and progesterone at E15 to control the timing of *NF1* loss led to optic glioma formation at 3 months (82), like those in *NF1+/-^GFAP^CKO* mice (77) and *BLBPCre*; *NF1^flox/mut^* mice (70). These studies confirmed that cell of origin was a determinant of optic glioma formation and that neuroglial progenitor cells, including GFAP+, BLBP+, CD133+ cells, and pre-oligodendrocyte precursor cells (pre-OPCs) that are Olig2+ and negative for NG2 could serve as initiating cells for murine NF1-OPG (70, 77, 82, 83). The spatial and cell type specificity of optic glioma formation resembled that of *KIAA1549-BRAF*-expressing pLGG model (66).

#### 4.2.2 NF1-LGG requires support of various cell types in the microenvironment for tumor growth and maintenance

In *NF1+/-^GFAP^CKO* mice, the formation of optic glioma only in *NF1+/-* mice with astroglial *NF1 *inactivation suggested that this tumor required microenvironment composed of cells heterozygous for *NF1 *mutation (77). Upon examination of the tumors in these mice, activated microglia in the tumor microenvironment was present (86). Daginakatte & Gutmann (2007) examined tumor specimens from human NF1-associated PAs and found microglia in all specimens (87). Microglia has been implicated in glioma as they were proposed to stimulate invasiveness of glioma (87, 100, 101). Daginakatte & Gutmann (2007) further found that *NF1^+/-^* brain microglia produced soluble factors, identified as hyaluronidase, that promoted *NF1^-/-^* astrocyte growth *in vitro* and *in vivo* (87). Since there were no low-grade glioma cell lines that simulated NF1-OPG, they utilized Adenovirus-Cre (Ad5-Cre) and *NF1^flox/flox^* astrocytes to generate *NF1^-/-^* astrocyte and microglia cultures (87). Microglia inactivation or genetic ablation consequently resulted in decreased optic glioma proliferation in *NF1+/-^GFAP^CKO* mice (87, 91). Furthermore, *NF1^+/-^* microglia exhibited increased c-Jun-NH2-kinase (JNK) pathway activation, without any significant changes in AKT, MAPK or p38-MAPK activity. Thus, JNK inhibition reduced proliferation, motility, and proinflammatory cytokine production of *NF1^+/-^* microglia, and inhibition of this pathway was sufficient to reduce optic glioma growth *in vivo* (88).

*NF1* heterozygosity was sufficient to increase microglia proliferation and motility *in vitro* and *in vivo* (88), but remarkably, this effect had temporal and spatial specificity (91). Simmons et al. (2011) demonstrated that *NF1* heterozygosity resulted in increase in microglia specifically within optic nerve, and not in brainstem or neocortex. Additionally, this increase in microglia numbers, which facilitated glial cell proliferation, occurred at a critical time during optic glioma development (91). Thus, they postulated that the increase in microglia in *NF1^+/-^* optic nerve likely resulted from defect in microglia homing and delay in dispersal of microglia from the optic nerve (91). Furthermore, the finding that CX3CR1-expressing stromal microglia were required for optic glioma formation in NF1-OPG mouse model established the role of microglia as essential drivers of optic gliomagenesis (93). Subsequent research demonstrated that *NF1* mutation resulted in higher expression of the cytokine Midkine that activated CD8+ T-cells, which then produced Ccl4, a cytokine that induced microglia to express Ccl5 necessary for glioma growth and formation (89, 90). These studies established the mechanisms by which *NF1* mutations affected the tumor microenvironment which contained various factors that regulated optic glioma growth.

## 5 Using hIPSCs to develop non-NF1 and NF1-pLGG xenograft models

A recent report by Anastasaki et al. (2022) utilized human induced pluripotent stem cells (hIPSCs) to generate LGG xenografts harboring *NF1* loss and *KIAA1549-BRAF* fusion. First, they engineered different hIPSC lines with patient-derived germline *NF1* mutations and with *KIAA1549-BRAF* fusion, then they differentiated these hIPSCs to multipotent human neural stem cells capable of generating both neuronal and glial lineage cells (hINPCs). These hINPC lines exhibited increased proliferation, and *KIAA1549-BRAF*-expressing hINPCs had increased MAPK pathway activation (68). Since injections into the mouse optic nerve, the most common site for NF1-pLGGs, caused a lot of tissue damage, *NF1-*null hINPCs were injected into the brainstem of immunocompromised *Rag1^-/-^* mice, which was the second most common site of NF1-pLGGs (68). Brainstem injections of *NF1-*null hINPCs and cerebellar injections of *KIAA1549-BRAF*-hINPCs formed LGGs at 1 month post injection. Moreover, these lesions exhibited many histopathologic features of human pLGGs. Mice with hIPSC-derived LGGs did not exhibit increased mortality, and the lesions had similar proliferative indices even though the sizes grew over time (68). These mirrored clinical observations in pLGG patients.

Orthotopic transplantation of *NF1-*null and *KIAA1549-BRAF*-expressing hIPSC-derived glial restricted progenitors (iGRPs) and oligodendrocyte progenitors (iOPCs), but not hIPSC-derived terminally differentiated astrocytes formed LGGs in *Rag1^-/-^* mice (68). This was reminiscent of the genetically engineered NF1-OPG mice in which neuroglial progenitor cells could serve as initiating cells of optic glioma formation (82, 92). Interestingly, iGRPs gave rise to tumors resembling optic pathway and brainstem gliomas, while iOPCs gave rise to tumors similar to many cerebellar human PAs (68).

Furthermore, Anastasaki et al. (2022) demonstrated that formation of LGGs required CD4+ T cell depletion and reduced astrocytic *Cxcl10* expression. So LGGs could form in *NOD/SCID*, CD4-deficient, CD4/CD8-deficient mice, but not in CD8-deficient mice or other strains lacking expression of microglia or T cell chemokine receptors (68). They also showed that primary human PA cell lines including that with an NF1-PA (JHH-NF1-PA) and that with sporadic PA (Res186) could form LGGs in *Rag1^-/-^* and *Cxcl10^-/-^* mice (68). Although additional work is still required to create preclinical models that more closely recapitulate patient tumors, this model provides an important milestone in the development of a humanized pLGG orthotopic xenograft model.

## 6 Zebrafish as models for pLGG

Another *in-vivo* model that has been utilized to study pLGGs is the zebrafish. It is cost-effective, has short experimental timeframe, and enables rapid investigation into tumor growth, invasion, metastasis, and drug screening (56, 102–105). Fewer cells are needed to establish tumor xenografts, and the availability of transparent transgenic zebrafish or their translucent larvae made it possible to track tumor cells in real-time (102–104, 106). Several transgenic zebrafish models have also been generated to interrogate glioma pathogenesis. For example, using the Gal4-UAS system, Mayrhofer et al. (2017) generated zebrafish brain tumor model that expresses oncogenes that activate MAPK and PI3K signaling in neural progenitor cells and discovered that activation of YAP signaling pathway promotes development of aggressive brain tumors (107). Luo et al. (2021) used CRISPR/Cas9 to establish transgenic zebrafish lines that express mutated *NF1*, *Rb1* or *TP53* under *GFAP* promoter and showed that various combinations of *NF1*, *TP53*, and/or *Rb1* mutations can induce gliomas of different grades and phenotypes (108). Using transgenic zebrafish, Lee et al. (2010) demonstrated that *NF1* knockdown increased ERK signaling and increased OPC proliferation in the developing spinal cord (109). Orthotopic xenografts and immunodeficient zebrafish models have also been developed for brain tumors (110–113). Although many of the zebrafish xenograft models used glioblastoma cells, Yuan et al. (2021) injected JHH-NF1-PA1 cells into the midline in the optic tectum of zebrafish at 2 days post fertilization, and they found that the pLGG cells survived over 6 days and migrated in the brains of larval zebrafish before their adaptive immune system matured (56). Sigaud et al. (2023) also used zebrafish embryos to evaluate therapeutic options for pLGG xenografts generated by injecting DKFZ-BT66 and BT40 cells (105). While zebrafish models certainly have advantages over mouse models in terms of time and cost efficiency and are useful for rapid drug screening, development of more models and/or a combination of different types of models will be necessary to recapitulate the complexity of human pLGGs.

## 7 Development of NF1 genetically engineered minipigs

Isakson et al. (2018) used Transcription activator-like effector nucleases (TALENs) flanking a known *NF1* nonsense mutation *NF1^R1947^* to transfect fetal Ossabaw minipig fibroblasts. NF1 minipigs were generated through chromatin transfer and subsequent breeding (114). *NF1* mutant allele exhibited germline transmission with Mendelian frequency and no evidence of reduced fitness (114). NF1 minipigs displayed features of NF1 patients such as skin abnormalities, neurofibromas, underwent biallelic inactivation of the *NF1* gene, and they also developed OPG (114). Therefore, the generation of these NF1 minipigs paved a path for the field to interrogate NF1-related molecular pathogenesis, explore therapeutic options, and conduct preclinical testing in large animal models that hopefully present closer resemblance to human patients.

## 8 Leveraging the strengths and overcoming the challenges of generating pLGG *in vitro* and *in vivo* models

Although massive number of efforts have been directed at developing preclinical *in vitro* and *in vivo* models of pLGGs, there are many challenges to these endeavors. The generation of *in vitro* pLGG models has been hampered by many factors. First, the lack of availability of patient tissues as some pLGGs located in less accessible areas such as NF1-OPGs are rarely biopsied (4, 7), although a few NF1-PA cell lines are available (115). Second, pLGG samples obtained from young children as patients are usually small, hence only small number of cells can be obtained to culture. Third, the intrinsic slow growth and benign behavior of these tumors coupled with OIS made it difficult to grow these tumor cells *in vitro* (48, 49, 52, 54, 60). Several methods were developed to bypass OIS through genetic modifications of the pLGG cells so that they can be propagated long enough in culture (53, 55), but these methods might generate *in vitro* model systems that incompletely reflect the genetic/epigenetic background of the primary tumors. Nevertheless, as more research is being done into developing different ways to extend the lifespan of pLGG cells *in vitro*, certain methods such as conditional reprogramming culture conditions or using hIPSC-derived hINPCs might provide plausible solutions to generating viable *in vitro* models (56, 68).

Two main groups of pLGG *in vivo* models have been generated by several labs with varying degree of success: patient-derived xenograft models of mostly non-NF1-pLGGs (50, 51, 60, 65, 66, 116) and genetically engineered mouse models mostly for NF1-pLGGs especially NF1-OPGs (70, 72, 76–79, 82). However, some have also attempted to generate NF1-pLGG patient-derived xenograft models (68, 83) and transgenic non-NF1-pLGG model (67).

Genetically engineered NF1-OPG models have been extremely useful to investigate the pathogenesis of NF1-OPG, which could somewhat be extended to general NF1-pLGGs. In these models, tumors were readily detectable as they arose in predictable locations with near 100% penetrance, the histopathological features of these tumors resembled those in the patients, the initiating event (*NF1* inactivation) was known, thus these models recapitulate many genetic and cellular abnormalities seen in NF1-OPG patients (94). Moreover, these tumors arose in immunocompetent mice allowing the interrogation into the tumor-immune axis that was necessary in NF1-OPG formation.

Nonetheless, there are many challenges presented to researchers in developing these pLGG *in vivo* models, and these reasons might vary depending on whether the pLGG is associated with NF1. For example, NF1-OPG formation depends on many factors including: temporal and spatial specificity (77, 78, 80, 91, 92), cell-of-origin (72, 76–78, 82, 83, 92), interaction with microenvironment (87–91, 93) and other signaling pathways (79, 84, 85, 117), nature of the *NF1* mutation (97), among others. Meanwhile, the challenges in generating non-NF1 pLGG models include the low proliferation capacity and the specific permissive tumor microenvironment necessary for glioma formation (7). Interestingly, NF1-OPG requires *NF1* heterozygous cells along with multiple different types of cells in the microenvironment to grow (77, 87–91, 93), whereas NF1 and non-NF1-pLGG xenografts needed loss of specific T-cell population to form (68). Consequently, the combination of patient-derived xenograft models and transgenic models are required to comprehensively interrogate the molecular pathogenesis of pLGGs and to assess potential immune response to therapeutic agents.

## 9 Future directions on pLGG modeling

Although most *in vitro* and *in vivo* models that were generated often expressed *BRAF* and *NF1* mutations, there are other genetic alterations that have been highlighted as hallmarks of certain types of pLGGs. Some reports proposed that *FGFR1* mutation may be a relevant prognostic marker in PAs as in some cases *FGFR1* mutations were associated with more adverse outcomes in patients (42). *FGFR1* hotspot mutations were also relatively frequent in pLGGs especially in mixed neuronal-glial tumors without known genetic drivers, providing an additional way to classify these tumors (118). Moreover, Egbivwie et al. (2019) generated 5 grade 1 PA patient-derived cell lines and found that *FGFR1* overexpression alone was able to increase tumor cell migration and drive tumor progression. They also found that there was higher expression of membranous phosphorylated FGFR1 in grade 2 tumors, so the presence of pFGFR1 could be associated with malignancy and tumor grade (119). Future research could add more focus into generating pLGG models that interrogate the pathogenesis of rarer alterations such as *FGFR*, *NTRK*, and *MYB* mutations. Development of pLGG models could also consider epigenetic events that might promote tumor survival, maintenance and/or progression as much less is known about the role of epigenetics in pathogenesis of pLGGs (4).

Due to the difficulties in generating long-term cultures of pLGG patient-derived cell lines, more alternative methods could be explored. For example, since there is such strong dependence of pLGG formation *in vivo* on microenvironment, certain factors could be added into the cultures such as cytokines or tumor cells could be co-cultured with other cell types to provide more supportive microenvironment. The relatively successful sECM method to culture pLGG cells demonstrates that elements in the microenvironment will be necessary to generate viable *in vitro* pLGG models (57). There is also a need to develop more sophisticated technologies for genetic manipulation of tissues and cells. Indeed, emerging somatic transgenic *in vivo* mouse (120–123) and 3D cerebral organoid-based human glioma models (124) genetically manipulated using electroporation also hold promise for more flexible and renewable modeling. These models can employ plasmid or mRNA-delivered transgenes, and/or CRISPR/Cas elements to enable more rapid recapitulation of mutations in diverse spatiotemporal contexts but have been mostly employed to generate HGG models (120–123).

**Table 5:** List of abbreviations commonly used in the manuscript.

Abbreviation	Definition
pLGG	Pediatric low-grade glioma
PA	Pilocytic astrocytoma
MAPK	Mitogen-activated protein kinase
ERK	Extracellular signal-regulated kinase
*KIAA1549*	A gene that is a common partner of *BRAF* in *BRAF* fusion-driven pediatric low-grade glioma
*BRAF*	B-raf proto-oncogene encoding a serine/threonine kinase protein
*QKI*	K-homology domain containing RNA binding gene encoding for the RNA-binding protein Quaking. This gene is a common fusion partner of *MYB* in pediatric low-grade glioma
SNV	Single nucleotide variant
JPA	Juvenile pilocytic astrocytoma
*CDKN2A*	Cyclin dependent kinase inhibitor 2A gene
NF1	Neurofibromatosis type 1, a genetic disease due to mutation in *NF1* gene. *NF1* gene encodes for the protein Neurofibromin 1.
FGFR1	Fibroblast growth factor receptor 1
*KRAS*	Kirsten rat sarcoma viral oncogene homolog. An oncogene that encodes small GTPase called KRAS.
H3F3A	H3 histone family member 3A
DA	Diffuse astrocytoma
PXA	Pleomorphic xanthoastrocytoma
RAS	Rat sarcoma virus, family of small GTPases
OPG	Optic pathway glioma
PI3K	Phosphatidylinositol 3-kinase protein
AKT	A serine/threonine kinase encoded by the oncogene in the transforming retrovirus isolated from the thymoma cell line AKT-8, derived from the stock A strain k AKR mouse. Also called protein kinase B.
TKDD	Tyrosine kinase domain duplication
TACC1	Transforming acidic coiled coil containing protein 1
RGNT	Rosette-forming glioneuronal tumor
EVN	Extraventricular neurocytoma
PLNTY	Polymorphous low-grade neuroepithelial tumor of the young
*MTOR*	Mechanistic target of rapamycin kinase gene encoding for the mTOR protein, a serine-threonine kinase
DNET	Dysembryoplastic neuroepithelial tumor
*MYB*	Myeloblastosis family of transcription factors
*MYBL*	*MYB* proto-oncogene like
HGG	High grade glioma
*NTRK*	Neurotrophic tyrosine receptor kinase gene
*TSC*	Tuberous sclerosis complex gene
RTK	Receptor tyrosine kinase
*ALK*	Anaplastic lymphoma kinase gene
*ROS1*	ROS proto-concogene 1 encoding for an orphan receptor tyrosine kinase
*TRKB*	Tropomycin receptor kinase B gene
*PDGFRA*	Platelet-derived growth factor receptor alpha gene
OIS	Oncogene-induced senescence
SASP	Senescence-associated secretory phenotype
PPTP	Pediatric preclinical testing program
SV40TAg	Simian vacuolating virus 40 large T antigen
hTERT	Human telomerase reverse transcriptase
WT	Wild-type
MEK	MAP kinase kinase, encoded by the gene *MAP2K1*
RCAS/Tv-a	Replication competent avian leukosis virus with splice acceptor/Tv-a
NSC	Neural stem cells
BLBP	Brain lipid binding protein
NG2	Neuron glia antigen 2
GFAP	Glial fibrillary acidic protein
PDOX	Patient derived orthotopic xenograft
o-GSC	Optic glioma stem cells
OPC	Oligondendrocyte progenitor cells
hIPSC	Human induced pluripotent stem cells
iNPC	hIPSC-derived neural progenitor cells
iGRP	hIPSC-derived glial restricted progenitors
iOPC	hIPSC-derived oligodendrocyte progenitors

A truly useful pLGG model would ideally recapitulate the molecular pathogenesis of human pLGGs along with the dependency of these tumors on the microenvironment milieu and the inevitable activation of the MAPK pathway. However, despite the constant challenges, many important milestones were achieved in the development of *in vitro* and *in vivo* pLGG models. The combination of advanced sequencing technologies and prognostic methods and *in vitro* and *in vivo* models should be utilized to comprehensively investigate the pathogenesis of this highly heterogeneous group of pediatric brain tumors and identify viable therapeutic options.

## Author’s note

After the completion of this review, a related work was published (125). **Table 5** lists the abbreviations commonly used in the manuscript.

## Author contributions

GY: Writing – original draft, Writing – review & editing. JB: Funding acquisition, Resources, Supervision, Writing – review & editing.
